# Dynamic multimodal survival prediction in multiple myeloma integrating gene expression, longitudinal laboratory measurements, and treatment history

**DOI:** 10.1093/bib/bbag475

**Published:** 2026-09-07

**Authors:** Shangru Jia, Artem Lysenko, Keith A Boroevich, Alok Sharma, Tatsuhiko Tsunoda

**Affiliations:** Laboratory for Medical Science Mathematics, Department of Computational Biology and Medical Sciences, Graduate School of Frontier Sciences, The University of Tokyo, 7-3-1 Hongo, Bunkyo-ku, Tokyo 113-0033, Japan; Laboratory for Medical Science Mathematics, Department of Biological Sciences, School of Science, The University of Tokyo, 7-3-1 Hongo, Bunkyo-ku, Tokyo 113-0033, Japan; RIKEN Center for Integrative Medical Sciences, 1-7-22 Suehiro-cho, Tsurumi, Yokohama 230-0045, Japan; Laboratory for Medical Science Mathematics, Department of Biological Sciences, School of Science, The University of Tokyo, 7-3-1 Hongo, Bunkyo-ku, Tokyo 113-0033, Japan; RIKEN Center for Integrative Medical Sciences, 1-7-22 Suehiro-cho, Tsurumi, Yokohama 230-0045, Japan; Centre for Sustainable Futures, The University of the South Pacific, Laucala Campus, Laucala Bay Road, Suva, Fiji; College of Informatics, Korea University, 145 Anam-ro, Seongbuk-gu, Seoul 02841, Republic of Korea; Laboratory for Medical Science Mathematics, Department of Computational Biology and Medical Sciences, Graduate School of Frontier Sciences, The University of Tokyo, 7-3-1 Hongo, Bunkyo-ku, Tokyo 113-0033, Japan; Laboratory for Medical Science Mathematics, Department of Biological Sciences, School of Science, The University of Tokyo, 7-3-1 Hongo, Bunkyo-ku, Tokyo 113-0033, Japan

**Keywords:** multiple myeloma, dynamic survival prediction, multimodal deep learning, DeepInsight

## Abstract

Prognostic stratification in multiple myeloma (MM) relies on staging systems fixed at diagnosis, discarding temporal information accumulated during treatment. We developed a dynamic multimodal framework that predicts residual overall survival from observation windows of 1–18 months post-diagnosis. The model integrates DeepInsight-transformed gene expression, longitudinal trajectories of 10 laboratory analytes, and treatment history through missingness-aware gated fusion. On the Multiple Myeloma Research Foundation (MMRF) cohort from the Relating Clinical Outcomes in Multiple Myeloma to Personal Assessment of Genetic Profile (CoMMpass) study (*n* = 752), five-fold-specific models trained on the development dataset achieved a mean concordance index (C-index) of 0.773 ± 0.024 and 1-year time-dependent area under the receiver operating characteristic curve (AUC) of 0.789 ± 0.021 on a common held-out CoMMpass validation split, outperforming the evaluated survival-learning baselines including DeepSurv, a Cox proportional hazards neural network, and random survival forests. Kaplan–Meier stratification showed significant separation at all primary landmarks (log-rank *P<.001*, hazard ratios 3.46–3.93). A distilled student model retaining only the DeepInsight gene expression representation and five baseline clinical features transferred to an independent microarray cohort (GSE24080, *n* = 507) without retraining, achieving a C-index of 0.672 and a time-dependent AUC at 1-year of 0.740, supporting cross-cohort transferability in a reduced-input setting. Interpretability analyses recovered ubiquitin-proteasome, endoplasmic reticulum (ER) stress, and Interferon Alpha Response signals consistent with established myeloma biology. These findings support the potential of dynamic multimodal modeling for longitudinal prognostic assessment in MM.

## Introduction

Multiple myeloma (MM) is a clonally heterogeneous plasma cell malignancy accounting for ~10% of hematologic cancers, with overall survival spanning months to over a decade among newly diagnosed patients [1, 2]. The International Staging System (ISS) and its revisions (R-ISS, R2-ISS) combine serum ${\beta}_2$-microglobulin (${\beta}_2$M), albumin (ALB), lactate dehydrogenase (LDH), and high-risk cytogenetic aberrations into ordinal stages that remain the clinical standard for risk classification [3–6]. However, these systems share a fundamental constraint: they assign patients to fixed categories at diagnosis and do not incorporate the longitudinal biomarker dynamics or treatment context that accumulate over the course of therapy.

Computational approaches to survival prediction in MM have pursued several directions. Random forest models combining clinical and RNA sequencing (RNA-seq) features on the Relating Clinical Outcomes in Multiple Myeloma to Personal Assessment of Genetic Profile (CoMMpass) cohort achieved C-indices near 0.78 [7], and the transformer-based Simultaneous Cancer Outcome Prediction Estimator (SCOPE) framework addressed joint event prediction across disease phases [8]. Deep learning survival methods have evolved from single-time-point architectures such as Cox-based DeepSurv [9] and the competing-risks model DeepHit [10] to longitudinal formulations including Dynamic-DeepHit [11]. In parallel, multimodal fusion methods in computational pathology—including Multimodal Co-Attention Transformer (MCAT) [12], Pathomic Fusion [13], PORPOISE [14], and pathway-aware multimodal transformers such as SurvPath [15]—have demonstrated that cross-modal integration can improve survival prediction [16]. However, these architectures operate at fixed time points on histopathology–transcriptomics pairs and do not address the temporal, multi-source data structure characteristic of longitudinal hematologic care.

A distinguishing feature of MM management is its prolonged, multi-phase treatment course, spanning induction, transplant eligibility assessment, consolidation, and maintenance over months to years [1, 2]. During this period, prognosis-relevant information accumulates continuously. Biomarker trajectories—including free light chain (FLC) kinetics, serum M-protein response, shifts in ${\beta}_2$M and LDH—reflect treatment response, residual disease burden, and emerging clonal dynamics in ways that a single diagnostic snapshot cannot capture. In practice, clinicians reassess prognosis at defined milestones such as induction completion, transplant decision, and maintenance initiation. Yet most existing computational models in MM are trained on features captured at a single time point and therefore cannot be easily updated as new observations become available. This motivates the development of a prediction framework that incorporates time-varying clinical data and revises prognosis estimates dynamically over the treatment course.

Two methodological ideas motivate the present study. First, the landmark prediction framework [17] enables dynamic risk estimation at successive time points using all previously observed data; we extend this paradigm by processing full longitudinal sequences through temporal encoders rather than treating covariates as fixed summary values at each landmark. Second, gene expression profiling has established prognostic value in MM through signatures such as the 70-gene expression profile (GEP70) [18] and the Erasmus Medical Center 92-gene signature (EMC-92) [19], but incorporating high-dimensional expression data into predictive models remains challenging because of the curse of dimensionality. DeepInsight [20] addresses this problem by restructuring tabular features into a 2D image representation that preserves local feature relationships, enabling convolutional neural networks to exploit spatial co-expression structure. Although this transformation has been applied to drug response prediction [21], it has not been explored in survival modeling. Separately, knowledge distillation [22] has been used to compress survival models [23] but has not been combined with multimodal dynamic prediction in hematologic malignancies. In MM, cohorts frequently provide only baseline expression data without longitudinal laboratory or treatment records. Compressing a multimodal model into a reduced-input student could facilitate deployment in settings where not all data modalities are available.

**Figure 1:**
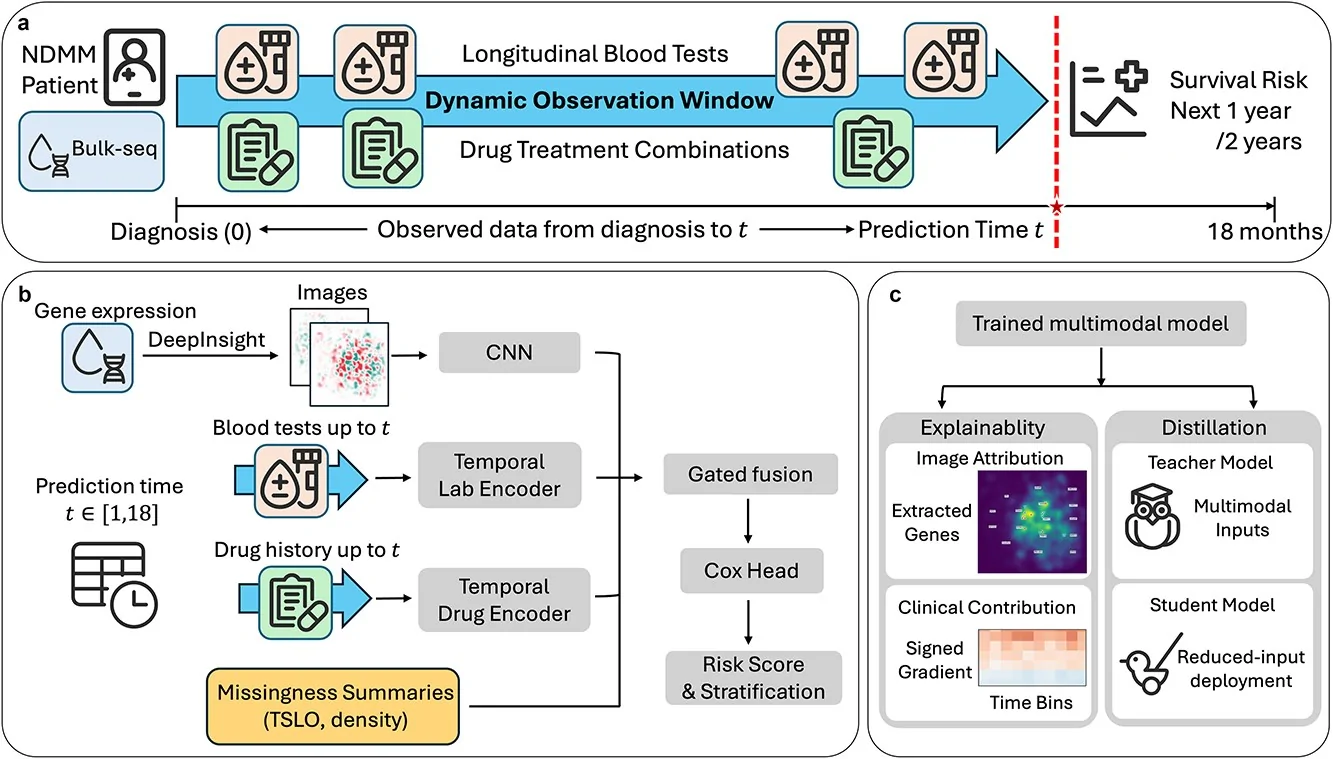
Study overview; (a) a newly diagnosed MM (NDMM) patient accumulates longitudinal blood tests and drug treatment records over a dynamic observation window from diagnosis to a prediction time *t* within the first 18 months postdiagnosis; the model estimates residual survival risk using all data observed up to *t*, together with a baseline DeepInsight-transformed gene expression representation; (b) model architecture: modality-specific encoders feed into missingness-aware gated fusion; a cox PH head outputs the log-hazard risk score, with auxiliary per-modality heads providing gradient supervision; (c) downstream applications: interpretability and teacher–student distillation for reduced-input external deployment.

Figure 1 summarizes the overall framework. The model integrates three data modalities (Fig. 1b)—gene expression image representations converted by DeepInsight, longitudinal laboratory trajectories, and treatment history—through gated fusion conditioned on observation reliability indicators, producing risk estimates from any observation window within the first 18 months post-diagnosis (Fig. 1a). We evaluate the framework on the Multiple Myeloma Research Foundation (MMRF) CoMMpass dataset using five-fold cross-validation, benchmark it against standard survival methods, validate it externally on the GSE24080 cohort using a distilled student model, and provide interpretability analyses linking model predictions to myeloma biology (Fig. 1c).

## Materials and methods

### Study design and cohort construction

The primary development cohort was drawn from the MMRF CoMMpass study (NCT01454297), a prospective longitudinal study of newly diagnosed MM patients with paired baseline bulk RNA-seq, serial laboratory measurements, and treatment records [24, 25]. Patients were included if they had (i) available baseline RNA-seq expression data, (ii) at least one post-diagnosis laboratory observation, and (iii) follow-up data sufficient to contribute to at least the earliest landmark (*t* = 1 month post-diagnosis). After applying these criteria, 752 patients were retained. The cohort of 752 patients was split by stratified random sampling on event status into a development set of 624 (124 deaths, 19.9% event rate; overall survival range 41–1984 days) and a held-out validation set of 128 (24 deaths, 18.8%), prior to any preprocessing. The development set was used for model training, hyperparameter tuning, and cross-validation. The held-out set was not used for parameter fitting, hyperparameter tuning, or checkpoint selection; it was reserved for post-training comparative evaluation of the fold-specific models and for selecting a representative fold for downstream analyses, as detailed in Supplementary Methods S1. Development and held-out validation sets were separated at the patient level prior to preprocessing or feature selection to prevent information leakage. Laboratory data spanned 18 monthly time bins across 10 myeloma-relevant analytes with 100% patient coverage; drug treatment records covered 74.4% of development set patients across three therapeutic classes.

For external evaluation, we used GSE24080 (UAMS-565), a publicly available microarray cohort of 507 MM patients profiled on Affymetrix U133Plus2 microarrays with available survival data (158 deaths, 31.1% event rate) [18, 26]. The higher event rate relative to CoMMpass reflects differences in treatment era, patient selection, and follow-up duration. Of the 5000 genes used in the development cohort, 4834 (96.7%) were matched by gene symbol in GSE24080; the remaining unmatched genes were zero-filled. Critically, GSE24080 provides only baseline gene expression and five summary clinical variables [hemoglobin (HGB), creatinine (CREAT), ALB, LDH, and ${\beta}_2$M]; the longitudinal laboratory time series and drug treatment records that constitute two of the three modalities in the full teacher model are not available. Despite this modality mismatch, the external evaluation is designed to test whether a distilled student model—retaining only the DeepInsight-transformed gene expression image and baseline clinical features—can transfer meaningful prognostic discrimination to an independent cohort measured on a different platform.

### Dynamic landmark prediction framework

The model operates in a dynamic landmark paradigm [17]: for a given patient and prediction time $t\in \left\{1,2,\dots, 18\right\}$ months post-diagnosis, it observes all available data up to *t* and predicts residual survival risk over the subsequent 1–2-year horizon. This formulation is motivated by the clinical workflow in MM, where risk reassessment occurs at defined treatment milestones rather than at a single fixed time point. Unlike phase-specific prediction approaches that model events within a particular disease phase [8], the landmark framework produces continuously updated risk trajectories conditioned on the accumulated longitudinal records and thus enables a single model to serve multiple clinical decision time points. This formulation ensures that predictions at each time point are based solely on information that would be available in a real clinical setting. A single architecture was used across all landmark times, with the current prediction time * t * encoded as a learned time embedding. Temporal integrity was enforced at the data level: the laboratory encoder received only time bins 1 through * t *, the treatment encoder received only pre-* t * drug records, and residual survival days were defined as $ os\_ days-t\times 30 $, where $ os\_ days $ denotes observed overall survival time in days from diagnosis. Only patients alive and not previously censored at time * t * contributed to evaluation at that landmark. Primary evaluation focused on * t=6 * and * t=12 * months.

### Data modalities and preprocessing

Each modality was selected to capture a distinct layer of prognostic information in MM: the gene expression representation encodes baseline tumor biology and molecular subtype; the longitudinal laboratory data capture evolving disease burden and treatment response; and the drug history provides treatment context. This complementarity is a key design premise—the longitudinal modalities were expected to capture more direct short-term disease dynamics, whereas the transcriptomic representation captures baseline biological state.

#### Gene expression feature representation

From the full transcriptome, 5000 genes were selected by variance-based filtering on the training set and transformed into 96 × 96 single-channel images using DeepInsight [20], with gene coordinates determined by *t*-distributed stochastic neighbor embedding (*t*-SNE) so that co-expressed genes map to spatially adjacent pixels. The resulting image introduces an inductive bias aligned with biological organization: convolutional filters can exploit local co-expression structure, in contrast to fully connected architectures that treat genes as independent features. Full preprocessing parameters (*t*-SNE perplexity, normalization, and percentile clipping) are provided in Supplementary Methods S1.

#### Longitudinal laboratory measurements

Ten analytes were selected for their established prognostic relevance in MM, including direct components of the ISS/R-ISS staging systems: HGB, CREAT, calcium, leukocytes, ALB, FLC lambda (FLC-*λ*), FLC kappa (FLC-*κ*), serum M-protein, LDH, and ${\beta}_2$M. Raw values were binned into monthly intervals, log-transformed where the distribution was right-skewed (FLC-*λ*, FLC-*κ*, LDH, and ${\beta}_2$M), and *z*-score normalized using means and standard deviations estimated only from the data used for model training. Missingness is an inherent feature of clinical laboratory data—sampling frequency varies by patient, institution, and clinical indication. To explicitly model this irregularity rather than treating it as nuisance, we accompany the observed values with binary observation masks $m\in \left\{0,1\right\}$ indicating whether each analyte was recorded in a given monthly bin and per-analyte time since last observation (TSLO). This design allows the model to explicitly distinguish between true absence of signal and missing observations, a critical property in real-world clinical data.

#### Treatment history

Drug exposure was encoded on the same monthly grid using three class-level binary indicators: bortezomib, carfilzomib, and immunomodulatory drugs (IMiDs). Each indicator takes the value 1 if the patient received any agent within that drug class during the corresponding monthly interval and 0 otherwise; dosage information was not used, as the CoMMpass dataset records drug administration at the regimen level without standardized dose fields. Only pre-*t* records were visible at prediction time *t* to avoid data leakage.

### Model architecture

The architecture follows a late-fusion multimodal design with a Cox proportional hazards (PH) prediction head (Fig. 1b). Each modality is first encoded independently before being combined through a gated fusion mechanism that dynamically weights their contributions.

#### Encoders and missingness-aware gated fusion

The DeepInsight-transformed gene expression image is encoded by a lightweight convolutional network; longitudinal laboratory trajectories and treatment history are encoded by separate Transformer modules with causal attention restricted to time bins ≤*t*, ensuring that no future information enters the prediction. The laboratory encoder is dual-stream: one stream embeds the observed analyte values, while a parallel stream embeds the missingness profile (binary observation masks and TSLO), so that the pattern of measurement is modeled jointly with the values themselves. Modality embeddings are then combined through a gated fusion module: a small gating network conditioned on the prediction time *t* and per-modality recency summaries produces softmax-normalized modality weights, and the fused embedding is the weighted sum of projected modality embeddings. Two regularization mechanisms address modality collapse: per-modality auxiliary Cox heads that supply direct gradient supervision to each encoder, and deploy-aware modality dropout that jointly zeroes the clinical streams during training (the image stream is never dropped), simulating reduced-input deployment in which only the baseline gene-expression-derived image representation is available. Layer dimensions, attention head counts, dropout rates, and auxiliary loss weights are reported in Supplementary Methods S1.

#### Prediction head and loss function

The fused representation is passed to the Cox PH head outputting a scalar log-hazard ratio (HR) ${h}_i$ for each patient *i*. The model is trained by minimizing the negative Cox partial log-likelihood:


$$ l=-\sum_{i:{\delta}_i=1}\left[{h}_i-\mathit{\log}\sum_{j\in R\left({t}_i\right)}\mathit{\exp}\left({h}_j\right)\right] $$

where ${\delta}_i$ denotes the event indicator and $R\left({t}_i\right)$ denotes the risk set at time ${t}_i$. The total training objective combines the fusion-level loss with auxiliary modality losses:


$$ L={l}_{fusion}+{\lambda}_{img}\cdotp{l}_{img}+{\lambda}_{tab}\cdotp{l}_{tab} $$

The complete teacher model contains ~1.6 million parameters.

The Cox formulation was chosen for its robustness to censoring and its compatibility with dynamic risk prediction settings.

### Training strategy

The training strategy was designed to address two practical challenges of the development cohort: limited sample size relative to model complexity, and an uneven distribution of events across landmark months, with later landmarks contributing fewer events and smaller risk sets. To ensure balanced patient representation and limit bias toward early follow-up, sampling was defined at the patient level so that each patient contributed a fixed number of landmark samples per epoch. Landmark months were sampled preferentially from earlier, event-rich periods at the start of training, and this preference was gradually relaxed to improve coverage across the full follow-up window. Five-fold stratified cross-validation was conducted within the development set. Training curves are shown in Supplementary Fig. S1, and complete implementation details are provided in Supplementary Methods S1.

### Knowledge distillation and external deployment

Typical cohorts rarely provide the complete set of longitudinal modalities available in MMRF CoMMpass. To enable deployment on such cohorts, the multimodal teacher was distilled into a compact student model [22]. The student (~1.0 million parameters) receives only the DeepInsight-transformed image of gene expression and five baseline clinical features: HGB, CREAT, ALB, LDH, and ${\beta}_2$M—selected for their availability in the target external cohort (GSE24080) and their prognostic roles in ISS/R-ISS staging [3–5]. This reduced-input design represents a deployment-oriented compromise: the teacher’s full multimodal knowledge is compressed into a model that operates in settings where longitudinal data are unavailable. The student image encoder was initialized from teacher weights. Training used a composite objective:


$$ {L}_{student}={\alpha}_{surv}\cdotp{l}_{Cox}+{\alpha}_{out}\cdotp{L}_{KD}+{\alpha}_{feat}\cdotp{L}_{align} $$

where ${l}_{Cox}$ is the standard Cox partial log-likelihood, ${L}_{KD}$ is output-level distillation matching teacher and student log-hazard predictions, and ${L}_{align}$ is feature-level cosine similarity. External evaluation on GSE24080 (*n=507*) proceeded without retraining; the 166 unmatched genes (of 5000) were zero-filled. This design enables practical deployment in settings where longitudinal data are unavailable while preserving key predictive signals learned by the full multimodal model.

### Interpretability

Model interpretability was assessed at three levels: (a) Integrated Gradients [27] on the image representation branch, with pixel attributions back-mapped to genes via pyDeepInsight coordinates and linked to pathways through pre-ranked Gene Set Enrichment Analysis (GSEA) and Over-Representation Analysis (ORA) against Molecular Signatures Database (MSigDB) Hallmark gene sets [28]; (b) gradient-based temporal attribution of laboratory values across the 18 monthly time bins; and (c) risk-stratified analysis of treatment-exposure patterns across model-defined risk groups. These analyses provide complementary insights into both molecular and temporal drivers of risk predictions.

### Evaluation metrics and benchmarks

Discrimination performance for survival risk prediction was assessed by Harrell’s concordance index (C-index) [29] and time-dependent area under the receiver operating characteristic curve (AUC) at 1-year and 2-year prediction horizons (tdAUC_1yr_, tdAUC_2yr_) computed via inverse probability of censoring weighting [30]. For survival risk stratification analysis, predicted risk scores were dichotomized into high- and low-risk groups using log-rank-optimized thresholds derived exclusively from the training set at each landmark; these thresholds were then applied without modification to the held-out validation and external cohorts. Kaplan–Meier survival curves were compared using the log-rank test, and HRs were estimated from Cox regression on the dichotomized groups. Benchmark methods spanned three categories reflecting distinct modeling paradigms: (1) classical penalized survival regression (elastic net Cox), (2) ensemble-based survival learning [random survival forest (RSF) [31], gradient-boosted survival analysis (GBSA)], and (3) deep survival networks, including DeepSurv [9], and a Cox-based multilayer perceptron (Cox-MLP). Each baseline was trained on three feature sets—clinical features, omics features reduced to 50 principal components by principal component analysis (PCA), and full concatenation—using matched cross-validation (CV) splits to separate the contribution of the learning algorithm from the input modality. This setup allows fair comparison across modeling paradigms while isolating the contribution of input modalities.

To assess clinical utility and to disentangle the temporal architecture from information availability, we additionally evaluated two further baseline families on the identical landmark-specific held-out risk sets. As clinical staging baselines we used the ISS stage and an available-component “ISS + baseline LDH” score, because the processed laboratory files do not contain assay-specific upper-limit-of-normal reference ranges, the latter was defined as a standardized additive combination of the ISS stage and the standardized baseline LDH value rather than a thresholded R-ISS criterion. Feasibility details for the unreconstructed R-ISS/R2-ISS, GEP70, and EMC-92 systems are provided in the Supplementary Methods (Section S1.6). As same-input dynamic survival baselines we used Landmark Cox, Landmark RSF, and Dynamic-DeepHit, each receiving the same longitudinal information available to the proposed model at each landmark. Differences in C-index were tested with a paired bootstrap (1000 resamples) on the patients for whom each baseline was defined.

## Results

### Cross-validation performance and landmark-wise evaluation

The proposed multimodal framework achieved strong and consistent performance across all evaluation settings, demonstrating robust survival discrimination. Five-fold-specific models trained within the development set (*n=624*) were evaluated on the same common held-out CoMMpass validation split, yielding a mean C-index of *0.773± 0.024*, tdAUC_1yr_ of *0.789± 0.021*, and tdAUC_2yr_ of *0.798± 0.034* (Supplementary Table S1). Performance was stable across folds (C-index range 0.733–0.806), indicating that the model learns stable representations despite the limited sample size and event rate. On the held-out validation set (*n=128*), the best model achieved C-indices of 0.793 at *t=6* months (*n=111*, 17 events) and 0.819 at *t=12* months (*n=99*, 10 events), with corresponding tdAUC_1yr_ of 0.813 and 0.818 (Supplementary Table S2). Bootstrap resampling (1000 replicates) yielded 95% confidence intervals of 0.713–0.882 at *t* = 6 and 0.709–0.913 at *t* = 12 (Supplementary Table S3); the wider interval at *t* = 12 reflects the small number of events at the later landmark and is discussed in relation to effective sample size in Section Limitations and future directions.

**Figure 2:**
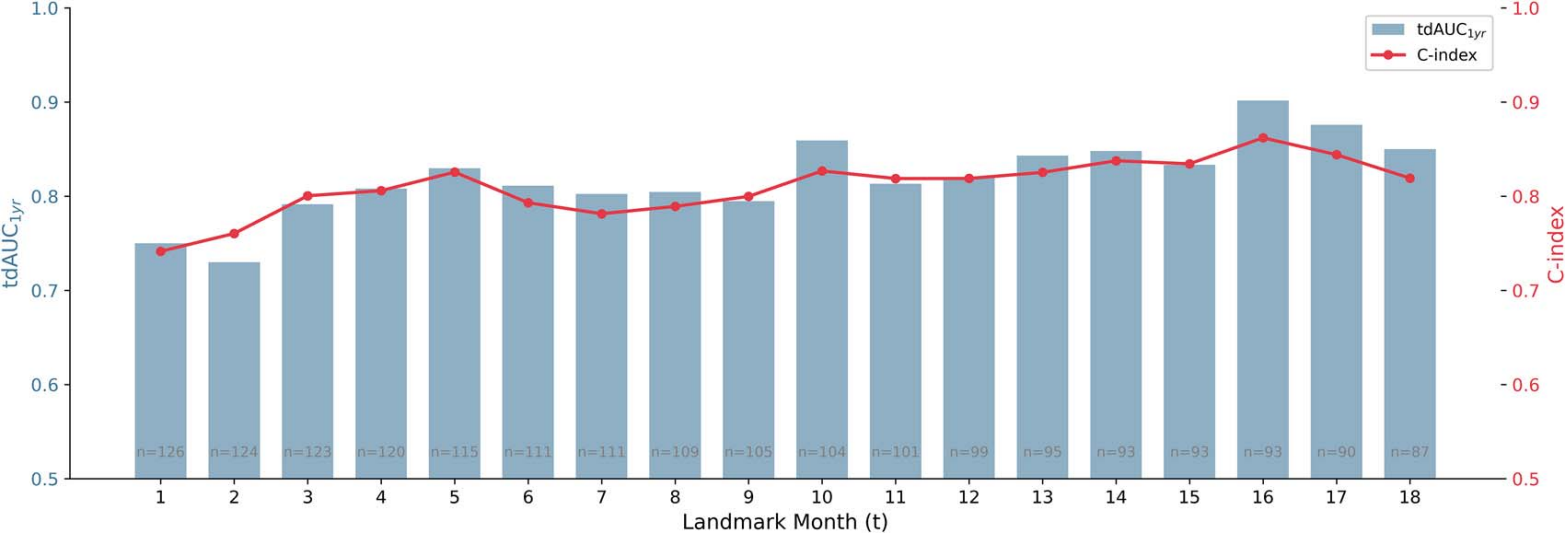
Landmark-wise evaluation across prediction months *t* = 1–18 on the held-out validation set (selected representative fold); bars: tdAUC_1yr_; line: C-index; sample sizes annotated at each landmark.

Landmark-wise evaluation across prediction months *t=1* through 18 is shown in Fig. 2. Both C-index and tdAUC_1yr_ remained in the 0.73–0.90 range, with discrimination increasing at later landmarks as additional longitudinal information became available. These results indicate that the model retained prognostic discrimination across the full range of observation windows rather than at a single favorable landmark.

### Benchmark comparison

**Figure 3:**
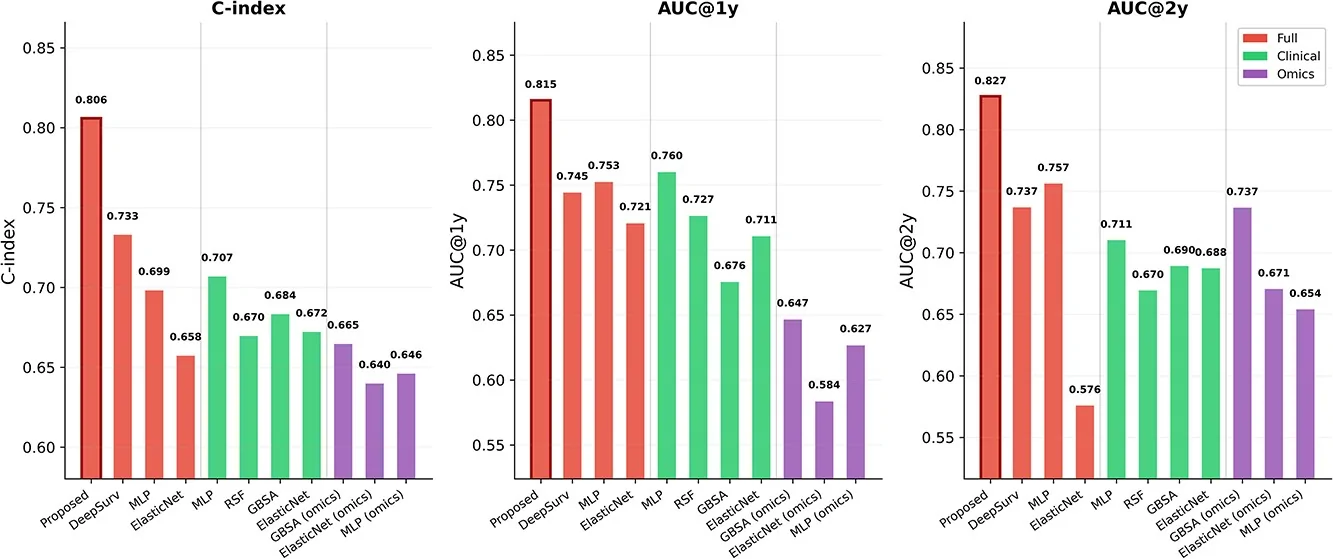
Benchmark comparison on the common held-out validation split, with each method represented by its fold-specific model with the highest held-out C-index; the proposed model and all baselines are compared across feature sets; AUC@1y and AUC@2y denote time-dependent AUC at the 1- and 2-year prediction horizons, respectively.

The proposed model consistently outperformed all baseline methods across all feature configurations and evaluation metrics. Figure 3 presents the best performance in the benchmark comparison on the held-out validation set; while cross-fold stability is reported in Table 1 and Supplementary Fig. S2. On the held-out validation set, it reached a C-index of 0.806, tdAUC_1yr_ of 0.815, and tdAUC_2yr_ of 0.827, with the strongest baseline (DeepSurv on the full feature set) at C-index of 0.733 (Supplementary Table S4).

**Table 1:** Benchmark comparison on the common held-out CoMMpass validation split.

Model	Features	C-index	tdAUC _1yr_	tdAUC _2yr_
Proposed	**Full**	**0.773** *±* **0.024**	**0.789** *±* **0.021**	**0.798** *±* **0.034**
DeepSurv	Full	0.633 *±* 0.095	0.656 *±* 0.098	0.634 *±* 0.098
Cox-MLP	Full	0.561 *±* 0.086	0.603 *±* 0.090	0.576 *±* 0.109
ElasticNet	Full	0.515 *±* 0.084	0.571 *±* 0.092	0.492 *±* 0.066
Cox-MLP	Clinical	0.626 *±* 0.097	0.643 *±* 0.100	0.618 *±* 0.105
RSF	Clinical	0.636 *±* 0.024	0.639 *±* 0.048	0.605 *±* 0.073
GBSA	Clinical	0.618 *±* 0.051	0.613 *±* 0.067	0.631 *±* 0.042
ElasticNet	Clinical	0.586 *±* 0.067	0.641 *±* 0.060	0.578 *±* 0.073
GBSA	Omics	0.578 *±* 0.085	0.530 *±* 0.108	0.650 *±* 0.076
ElasticNet	Omics	0.585 *±* 0.038	0.527 *±* 0.047	0.610 *±* 0.046
Cox-MLP	Omics	0.548 *±* 0.058	0.505 *±* 0.076	0.594 *±* 0.056

Values are mean *±* SD across models obtained from five-fold cross-validation on the development set. Best performance in bold.

Averaged across the five-fold-specific models on the common held-out split, the proposed model achieved a mean C-index of *0.773± 0.024*, compared with the next-best RSF at *0.636± 0.024* and DeepSurv at *0.633± 0.095*. The proposed model exhibited lower cross-fold variability (SD 0.024) than most deep learning baselines (DeepSurv SD 0.095; Cox-MLP SD 0.086–0.097), suggesting more stable learning.

### Overall survival risk stratification

**Figure 4:**
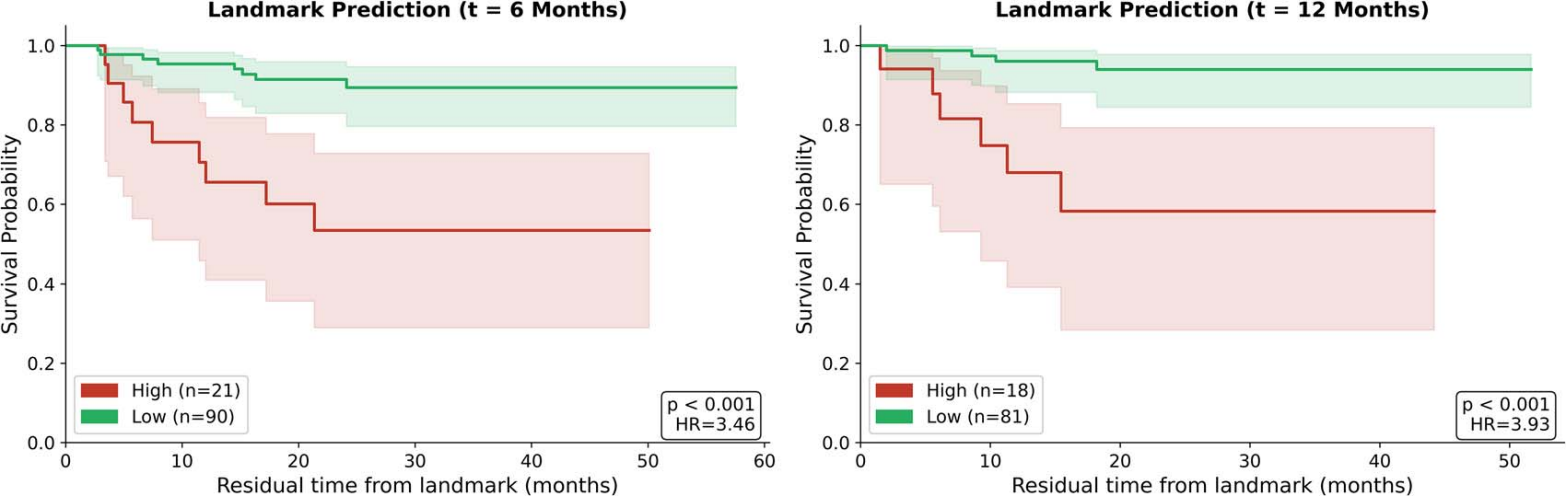
Kaplan–Meier survival curves stratified by model-predicted overall survival risk at landmarks *t* = 6 months (left) and *t* = 12 months (right) on the held-out validation set; shaded bands: 95% confidence intervals; HRs and log-rank *P*-values annotated.

Kaplan–Meier analysis on the held-out validation set showed significant survival risk-group separation at both primary landmarks (Fig. 4). At *t=6* months, the high-risk group (*n=21*) had lower survival than the low-risk group (*n=90*), with a HR of 3.46 (log-rank *P<.001*). At *t=12* months, separation was more pronounced (HR *=3.93*, *P<.001*; high-risk *n=18*, low-risk *n=81*). The larger HR at later landmarks suggests improved risk separation as more longitudinal information becomes available. These results demonstrate that the model’s predictions support clinically meaningful patient stratification. In addition, Kaplan–Meier-based grouped calibration at the 12-month landmark indicated that predicted and observed survival probabilities were broadly consistent, with mean absolute errors of 0.118 for the 1-year horizon and 0.088 for the 2-year horizon (Supplementary Fig. S3).

### Cross-platform evaluation of the distilled reduced-input model

To evaluate cross-platform transferability in a reduced-input setting, we evaluated a distilled version of the model (Materials and Methods) on an independent external cohort without retraining. The distilled student model was applied to GSE24080 (*n=507*, 158 deaths). It is important to note that this evaluation tests not the full teacher model’s generalizability but the viability of a reduced-input deployment path: whether a distilled model retaining only baseline features can transfer meaningful prognostic discrimination to an independent cohort measured on a different platform.

**Figure 5:**
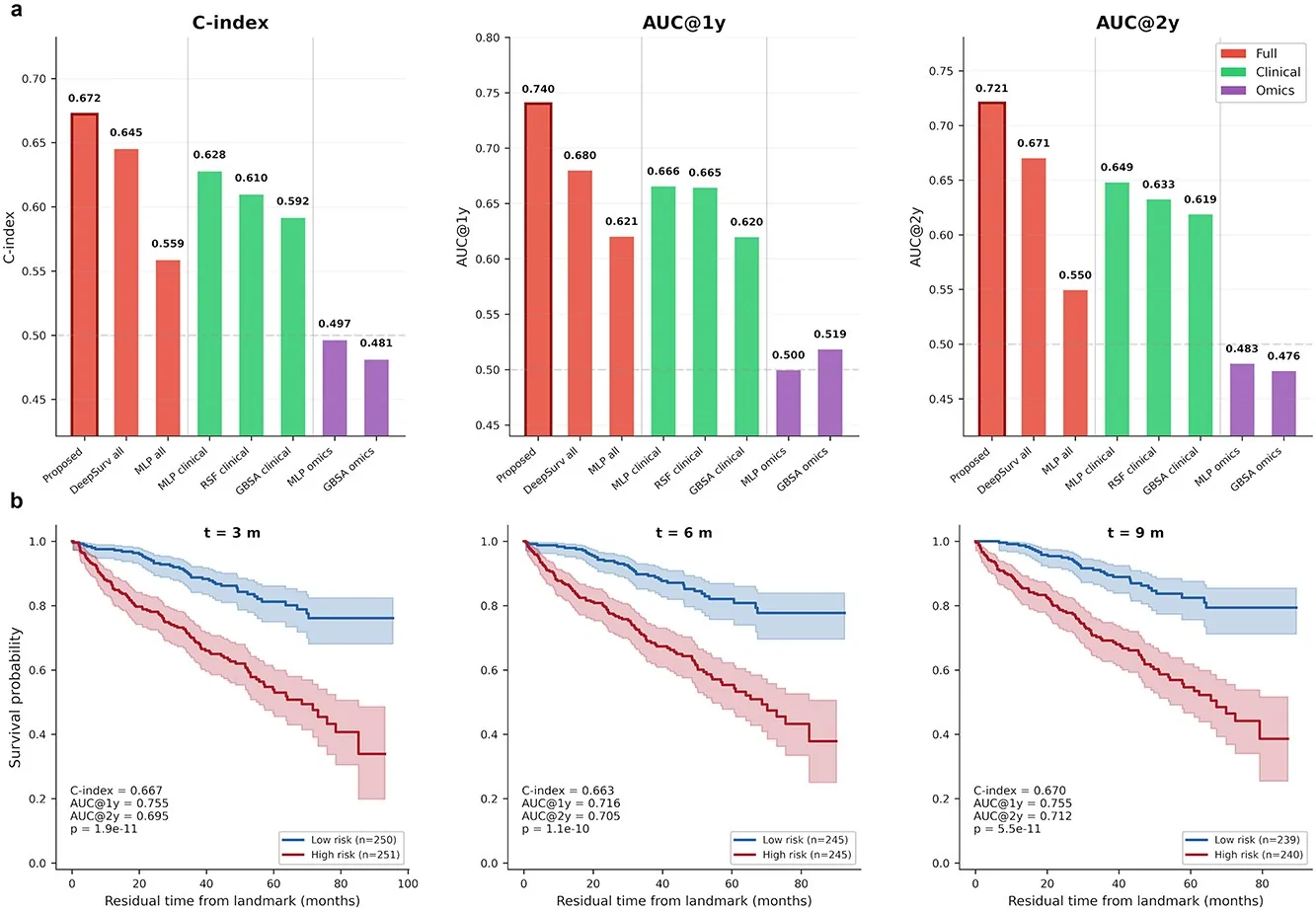
External reduced-input evaluation on GSE24080 (*n=507*); (a) benchmark comparison at *t* = 1 month, reporting C-index and time-dependent AUC at the 1- and 2-year horizons (AUC@1y and AUC@2y); (b) Kaplan–Meier survival curves at representative landmarks *t* = 3, 6, 9 months (m); C-index, AUC@1y, AUC@2y, and log-rank *P*-values are annotated; complete multi-landmark results are provided in Supplementary Table S6.

For this external reduced-input evaluation, each benchmarked model was represented by the fold-specific model selected on the common held-out CoMMpass validation split, and these selected models were then applied to GSE24080 without retraining or adaptation. At the baseline landmark (*t=1* month), the student achieved C-index of 0.672 and tdAUC_1yr_ of 0.740, outperforming all transferred baselines: DeepSurv (C-index 0.645), Cox-MLP clinical (0.628), RSF clinical (0.610), and GBSA clinical (0.592) (Fig. 5a and Supplementary Table S5). Multi-landmark evaluation showed stable performance from *t=1* through 12 months (C-index 0.649–0.672, tdAUC_1yr_ 0.710–0.755; Supplementary Table S6). Figure 5b shows representative Kaplan–Meier stratification at landmarks *t=3,6,9* months, all demonstrating significant risk-group separation (log-rank $P<{10}^{-10}$). These results support the feasibility of cross-platform transfer for the distilled reduced-input model.

### Comparison with clinical staging and dynamic survival baselines

To assess the contribution of the temporal architecture beyond information availability and to compare it against standard clinical staging, we evaluated the proposed model against same-input dynamic survival baselines (Landmark Cox, Landmark RSF, and Dynamic-DeepHit) and against the ISS staging score on the identical held-out landmark-specific risk sets (Table 2); an additional available-component ISS + baseline LDH comparison is provided in Supplementary Table S7.

**Table 2:** Comparison with clinical staging and dynamic survival baselines at the primary landmarks.

Comparator	Landmark	*n*	C-index (proposed)	C-index (comparator)	Delta-C	95% CI	*P*
ISS	*t* = 6	110	0.799	0.636	+0.163	0.062–0.295	0.006
ISS	*t* = 12	98	0.823	0.739	+0.084	−0.012 to 0.195	0.080
Landmark Cox	*t* = 6	111	0.793	0.607	+0.186	0.050–0.344	0.006
Landmark Cox	*t* = 12	99	0.819	0.579	+0.239	0.063–0.441	0.020
Landmark RSF	*t* = 6	111	0.793	0.690	+0.103	−0.060 to 0.284	0.224
Landmark RSF	*t* = 12	99	0.819	0.624	+0.195	0.029–0.382	0.034
Dynamic-DeepHit	*t* = 6	111	0.793	0.568	–	–	–
Dynamic-DeepHit	*t* = 12	99	0.819	0.538	–	–	–

C-indices were computed on matched held-out risk sets; ΔC denotes the difference from the proposed model, with *P*-values from paired bootstrap testing. Dynamic-DeepHit is reported for discrimination only. The available-component ISS + baseline LDH comparison is provided in Supplementary Table S7.

The proposed model achieved the highest C-index of all clinical and dynamic baselines at both primary landmarks (Table 2). Because the dynamic baselines receive the same time-resolved inputs, these comparisons isolate the contribution of the architecture rather than differences in information availability. The model was significantly more discriminative than Landmark Cox at both landmarks (ΔC = 0.186, *P* = .006 at *t* = 6; ΔC = 0.239, *P* = .020 at *t* = 12) and than Landmark RSF at *t* = 12 (ΔC = 0.195, *P* = .034), with a consistent advantage at *t* = 6 (ΔC = 0.103, *P* = .224); Dynamic-DeepHit was substantially weaker at both landmarks (C-index 0.568 and 0.538). This consistent advantage over both a linear (Landmark Cox) and a tree-based (Landmark RSF) dynamic baseline indicates that the temporal encoder and gated fusion capture time-dependent structure beyond simpler landmark models.

Against ISS staging, the model was significantly more discriminative at *t* = 6 (ΔC = 0.163, *P* = .006) and remained directionally superior at *t* = 12 (ΔC = 0.084, *P* = .080), the later landmark being limited by its smaller event count. A descriptive combined-landmark summary showed the same direction of improvement over ISS and the dynamic baselines (Supplementary Table S8), though this pooled analysis should be interpreted cautiously because the landmark-specific risk sets are not independent. Together with the modality ablation (Section Modality ablation), in which longitudinal laboratory trajectories were the strongest single modality, these results indicate that the model’s advantage derives largely from dynamically integrating longitudinal laboratory information rather than from any single static predictor.

### Modality ablation

Single modality ablation (Table 3 and Supplementary Figs S4 and S5) quantified each data source’s contribution and revealed a clinically interpretable hierarchy. Longitudinal laboratory measurements were the strongest individual modality (C-index *0.693± 0.021*, tdAUC_1yr_  *0.739± 0.026*), consistent with the centrality of ${\beta}_2$M, ALB, and LDH to ISS/R-ISS staging [3–5] and reflecting the fact that longitudinal trajectories capture the most direct near-term disease-state signal. Drug history alone reached C-index of *0.628± 0.045*; the relatively weaker standalone performance is expected, as treatment intensity correlates with disease severity (indication bias), making drug features more informative as a complement to clinical data than as an independent predictor. The DeepInsight image representation branch (*0.624± 0.029*) outperformed a flat expression MLP model on the same 5000 input genes (*0.596± 0.044*; $\Delta \mathrm{C}\approx 0.028$), supporting the value of the spatial co-expression inductive bias.

**Table 3:** Modality ablation (five-fold CV, mean ± SD).

Configuration	C-index	tdAUC _1yr_	ΔC
**Full (proposed)**	**0.773** *±* **0.024**	**0.789** *±* **0.021**	–
Lab only	0.693 *±* 0.021	0.739 *±* 0.026	*-* 0.080
Drug only	0.628 *±* 0.045	0.625 *±* 0.060	*-* 0.145
Expression (DeepInsight image representation)	0.624 *±* 0.029	0.590 *±* 0.030	*-* 0.149
Expression MLP	0.596 *±* 0.044	0.575 *±* 0.042	*-* 0.177

The full multimodal model improved over the best single modality by an absolute 0.080 in C-index (10.4% relative), indicating that the three data sources carry complementary prognostic information that the gated fusion mechanism successfully combines.

A complementary no-treatment-branch ablation, in which the model was retrained with the treatment encoder removed (image and laboratory branches only), changed discrimination only marginally and non-significantly (ΔC = +0.016, *P* = .716 at *t* = 6; ΔC = +0.052, *P* = .222 at *t* = 12), indicating that treatment history contributes prognostic context rather than acting as the dominant driver of held-out performance.

### Molecular and clinical interpretability

**Figure 6:**
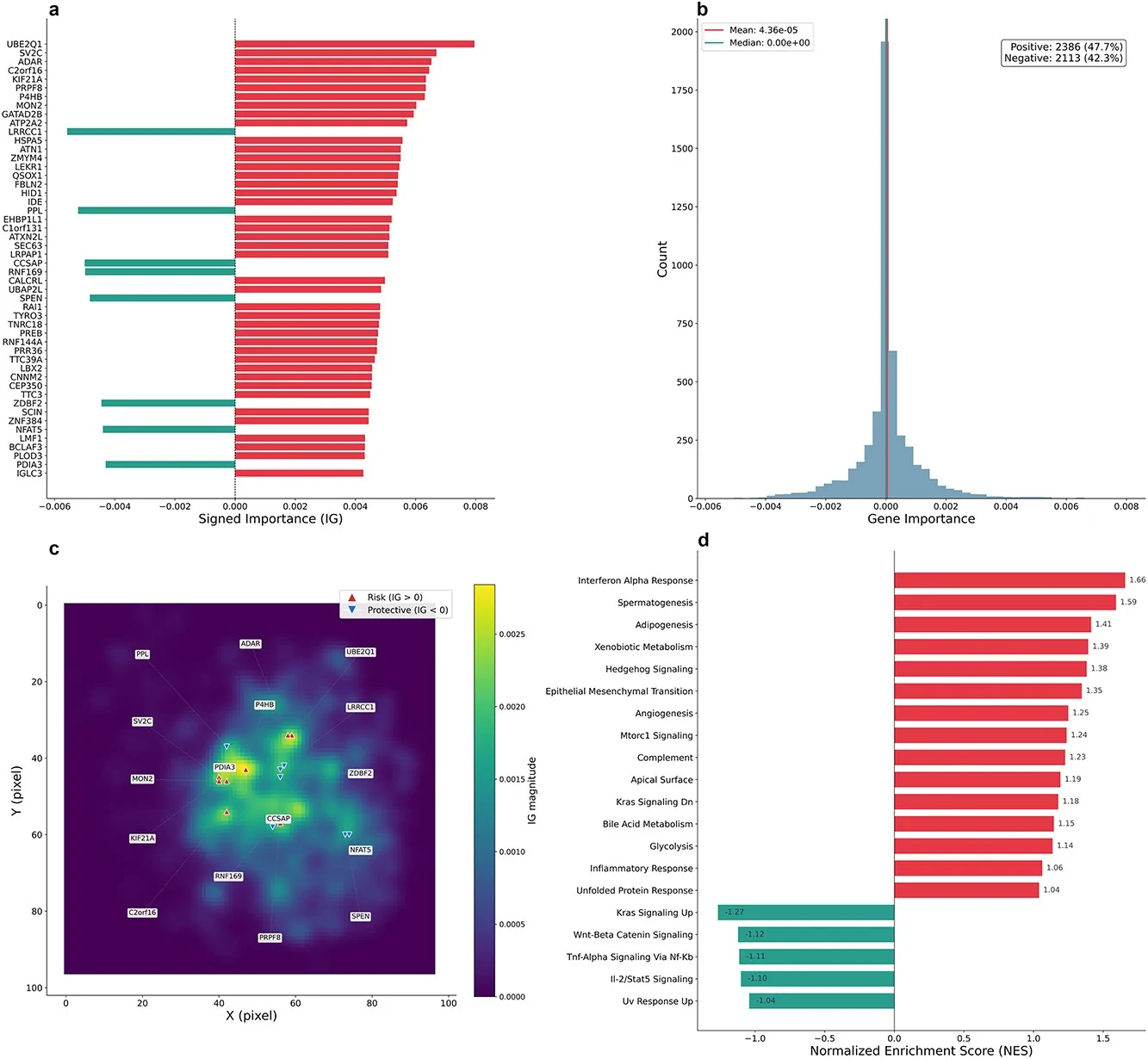
Gene-level interpretability; (a) top genes ranked by signed Integrated Gradients attribution; (b) distribution of gene-level importance scores; (c) spatial importance map on DeepInsight coordinates; (d) pathway enrichment via pre-ranked GSEA on MSigDB Hallmark gene sets.

The model’s predictions were examined for biological interpretability. Integrated Gradients on the gene expression image representation branch produced pixel-level attribution maps that were reverse-mapped to individual genes via pyDeepInsight coordinates (Fig. 6a–c). The top risk-associated genes included ubiquitin conjugating enzyme E2 Q1 (UBE2Q1) (ubiquitin-proteasome system), adenosine deaminase RNA specific (ADAR) (RNA editing), prolyl 4-hydroxylase subunit beta (P4HB) (endoplasmic reticulum stress), and pre-mRNA processing factor 8 (PRPF8) (spliceosome)—all with established relevance to myeloma biology, including pathways linked to proteasome inhibitor response [32–36]. Spen family transcriptional repressor (SPEN) (transcriptional regulation) and ring finger protein 169 (RNF169) (DNA damage repair) were identified in the attribution analysis as protective-associated genes, with higher expression associated with lower predicted risk. A gene-by-gene discussion with primary references is provided in Supplementary Discussion. Representative attribution maps stratified by risk group are shown in Supplementary Fig. S6, illustrating the spatial concentration of high-magnitude attributions in the high-risk group relative to the diffuse low-risk pattern.

Pre-ranked GSEA on MSigDB Hallmark gene sets (Fig. 6d; Materials and Methods) identified risk-associated enrichment in Interferon Alpha Response, consistent with evidence that type I interferon signaling can promote myeloma-associated immunosuppression and disease progression [37]. ORA on both risk-increasing and protective gene sets (Supplementary Figs S7 and S8; Materials and Methods) also identified Unfolded Protein Response and mTORC1 Signaling among risk-enriched terms. These associations support biological plausibility but do not establish causal relationships; the model captures correlative transcriptomic patterns that align with known myeloma biology rather than identifying therapeutic targets.

**Figure 7:**
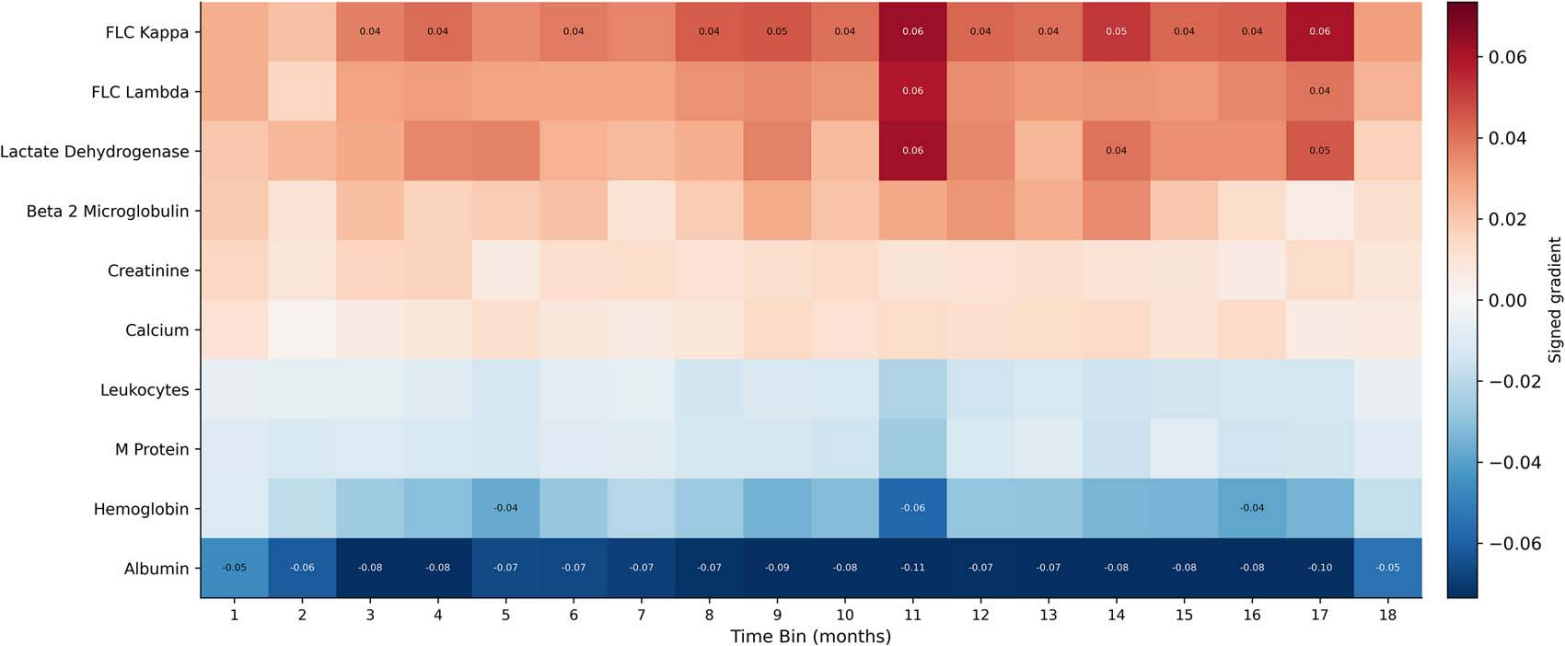
Laboratory temporal attribution heatmap; each cell shows the mean signed gradient ∂(log-hazard)/∂(analyte value) at the corresponding monthly time bin; positive values indicate that higher analyte values increase predicted mortality risk; negative values indicate a protective association; overall attribution patterns remain broadly consistent across months.

Temporal attribution of laboratory values was computed by backpropagating the model’s log-hazard output through the longitudinal-laboratory encoder and recording the signed gradient with respect to each observed analyte value at each monthly time bin. For a given analyte at a certain time bin, a positive gradient indicates that increasing the observed value at that time point would raise the predicted mortality risk, while a negative gradient indicates a protective association. The resulting heatmap (Fig. 7) revealed consistent direction structure across analytes: FLC-$\mathrm{\kappa}$, FLC-$\mathrm{\lambda}$, LDH, and ${\mathrm{\beta}}_2$M exhibited positive gradients throughout the observation window, confirming their established associations with disease burden. ALB showed the strongest negative (protective) gradients, followed by HGB—consistent with the known prognostic roles of these markers in ISS/R-ISS staging [3–5].

To characterize treatment context, we examined drug administration patterns stratified by model-predicted risk group (Supplementary Fig. S9). Patients in the high-risk group (death rate 34%) received more intensive treatment than those in the low-risk group (death rate 1%): carfilzomib and IMiD usage rates were consistently higher across all monthly time bins, with the largest differences emerging after Month 5. This pattern is consistent with clinical practice, where higher-risk patients are more likely to receive second-line proteasome inhibitors and combination regimens.

## Discussion

### Performance and dynamic prediction

We developed a multimodal deep learning framework for dynamic survival prediction in MM that integrates image representation of gene expression, longitudinal laboratory measurements, and treatment context through gated fusion. The model produces updated survival risk estimates from any observation window within the first 18 months of diagnosis while enforcing temporal integrity. Together, these features enable clinically aligned, time-adaptive risk prediction that extends beyond conventional static prognostic models.

The internal C-index of 0.806 is comparable to previously reported values on the CoMMpass cohort: Orgueira *et al*. [7] achieved 0.780 with random forests combining clinical and RNA-seq features, and Hussain *et al*. [8] reported 0.70–0.75 with SCOPE. Direct comparison across studies is confounded by differences in feature sets, cohort definitions, and evaluation protocols. Beyond such differences in datasets, a key distinction lies in the modeling paradigm: prior approaches relied on static or phase-specific features, whereas the proposed framework incorporates time-evolving clinical information within a unified dynamic model. This design enables the model to capture treatment response dynamics and evolving risk profiles that fixed-time-point architectures cannot access. We emphasize that the external evaluation on GSE24080 assessed only the reduced-input distilled student, because that cohort lacks the longitudinal laboratory and treatment modalities; the full longitudinal multimodal framework therefore remains externally unvalidated (Section Limitations and future directions).

### Limitations and future directions

This study has several limitations. The generalizability of the proposed framework across populations with different demographic profiles, treatment patterns, and follow-up structures remains to be determined. Although internal five-fold cross-validation supported stable performance within the CoMMpass cohort, external assessment on GSE24080 was conducted using a reduced-input student model because that cohort lacks longitudinal laboratory measurements and treatment histories. Consequently, the current external results provide limited evidence regarding cross-cohort generalizability of the full framework. Further evaluation in independent cohorts with harmonized multimodal data collection and longitudinal follow-up will be required to determine its utility in longitudinal clinical settings. In addition, the model was trained using the Cox partial likelihood, which relies on the PH assumption; this assumption may not hold in certain clinical subgroups, including patients with late-relapsing MM. Future studies should therefore investigate alternative survival modeling objectives, including rank-based losses and discrete-time formulations, to assess whether they provide greater robustness under non-PH settings. Several further points should be noted. Because the effective supervision in survival modeling is governed by the number of events rather than the total sample size, the held-out landmark-specific estimates rest on few events (17 at *t* = 6 and 10 at *t* = 12) and carry wide bootstrap intervals (Supplementary Table S3); they should be read together with the mean across the five-fold-specific models on the common held-out split (C-index 0.773 ± 0.024) and the repeated multi-seed result (0.764 ± 0.013, Supplementary Table S9), and the 1.6 M-parameter model is regularized by per-modality auxiliary heads and deploy-aware modality dropout to mitigate over-fitting at this scale. Parameter-sensitivity and calibration analyses further indicate that this added capacity does not translate into over-fitting, with discrimination remaining stable across a range of hyperparameter settings and predicted risks well calibrated across the cross-validation folds (Supplementary Tables S10 and S11). Schoenfeld residual tests at the primary landmarks showed no significant evidence of a proportional-hazards violation (global *P* = .52 and .85; Supplementary Table S12), although the small event counts make this a diagnostic rather than a definitive check. Treatment exposure is encoded as monthly class-level binary indicators (bortezomib, carfilzomib, and IMiDs) without dosage or duration, because CoMMpass records administration at the regimen level without standardized dose fields; incorporating dose, duration, and line of therapy is left for future work, and because treatment intensity is confounded with disease severity the treatment signal should be interpreted as prognostic context rather than a causal effect.

## Conclusion

We developed a dynamic multimodal survival prediction framework for MM that integrates gene expression representations, longitudinal laboratory trajectories, and treatment history through gated fusion. The framework achieved strong and consistent performance: on a common held-out CoMMpass split, the five-fold-specific models achieved a mean C-index of *0.773± 0.024* and tdAUC_1yr_ of *0.789± 0.021*. A distilled, reduced-input student model retained discriminative power on an independent external cohort (C-index of 0.672, tdAUC_1yr_ of 0.740), despite platform differences and restricted inputs. Because that cohort lacked longitudinal laboratory and treatment data, the external evaluation assessed the reduced-input student rather than the complete longitudinal multimodal model. Interpretability analyses identified molecular and clinical patterns consistent with known myeloma biology, supporting the biological plausibility of the model’s predictions. Overall, these results support the potential of dynamic multimodal frameworks to improve prognostic modeling in longitudinal clinical settings such as MM. Future work will focus on prospective validation with complete multimodal data through clinical collaboration to assess translational utility.

**Key Points**

- This study presents a dynamic multimodal deep learning framework for overall survival prediction in multiple myeloma, enabling risk estimation to be updated over observation windows within the first 18 months after diagnosis.
- The framework integrates DeepInsight-transformed gene expression, longitudinal trajectories of 10 laboratory analytes, and treatment history through temporally constrained encoders and a missingness-aware gated fusion mechanism.
- On the Multiple Myeloma Research Foundation (MMRF) cohort from the Relating Clinical Outcomes in Multiple Myeloma to Personal Assessment of Genetic Profile (CoMMpass) study, the framework outperformed benchmarked baselines, with a mean concordance index of 0.773 and a 1-year time-dependent area under the receiver operating characteristic curve (AUC) of 0.789 on the common held-out validation split, and showed significant separation between predicted survival risk groups.
- A reduced-input distilled student model was evaluated on the independent GSE24080 microarray cohort without retraining, retaining discriminative performance under restricted-input conditions.
- Interpretability analyses identified transcriptomic and clinical signals consistent with established myeloma biology, and the modular code and reproducibility resources are publicly available to support reuse.

## Supplementary Material

supplementary_file_bbag475

## Data Availability

The primary development cohort was derived from the MMRF CoMMpass study [24, 25], available via the MMRF Researcher Gateway subject to data-access requirements. External evaluation data are publicly available from the NCBI Gene Expression Omnibus (GEO) under accession GSE24080 [26]. A reproducibility package containing preprocessed data and training outputs is archived on figshare: https://doi.org/10.6084/m9.figshare.31804762.

## Code availability

The source code has been modularized and made publicly available at the GitHub repository: https://github.com/shangruJia/multi_mm.
