# Supplementary material for: Dynamic multimodal survival prediction in multiple myeloma integrating gene expression, longitudinal laboratory measurements, and treatment history
Source: Brief Bioinform. 2026 Sep 7;27(5):bbag475. doi: 10.1093/bib/bbag475 (PMC13548322; doi:10.1093/bib/bbag475)
Supplement: Supplementary file 1 [file supplementary_file_bbag475.docx]

**Supplementary Materials**

*Dynamic multimodal survival prediction in multiple myeloma integrating gene expression,*

*longitudinal laboratory measurements, and treatment history*

**Supplementary Tables**

**Table S1:** Performance of the five fold-specific models evaluated on the common held-out CoMMpass validation split ($n=128$). Each model was trained on a different four-fold partition of the 624-patient development set; the held-out split was not used during training in any fold.

| **Fold** | **C-index** | **tdAUC_1yr_** | **tdAUC_2yr_** | **tdAUC_1yr_**  **at** $\boldsymbol{t=6}$ | **Status** |
| --- | --- | --- | --- | --- | --- |
| 0 | 0.776 | 0.812 | 0.791 | 0.861 | Completed |
| 1 | 0.780 | 0.774 | 0.811 | 0.775 | Completed |
| 2 | 0.806 | 0.815 | 0.826 | 0.813 | **Selected** |
| 3 | 0.733 | 0.778 | 0.735 | 0.741 | Completed |
| 4 | 0.770 | 0.764 | 0.827 | 0.758 | Completed |
| Mean ± SD | 0.773 ± 0.024 | 0.789 ± 0.021 | 0.798 ± 0.034 | 0.790 ± 0.043 | — |

**Note.** Five-fold stratified cross-validation was performed on the 624-patient development set. The resulting fold-specific models were each evaluated on the common held-out split ($n=128$); the mean ± SD summarizes the variation across these five models. Fold 2 was selected as the representative fold for downstream analyses (landmark-wise plots, Kaplan–Meier stratification, and external transfer experiments) based on the highest mean tdAUC_1yr_ across the primary landmarks ($t=6,12$ months) on this held-out split.

**Table S2:** Held-out validation performance at primary landmarks for the selected fold (fold 2, n = 128). Metrics were computed using inverse-probability-of-censoring weighting.

| **Landmark** | **n** | **Events (%)** | **C-index** | **tdAUC_1yr_** | **tdAUC_2yr_** |
| --- | --- | --- | --- | --- | --- |
| $t=6$ months | 111 | 17 (15.3%) | 0.793 | 0.813 | 0.816 |
| $t=12$ months | 99 | 10 (10.1%) | 0.819 | 0.818 | 0.837 |

**Note.** Aggregate: tdAUC_1yr_ avg = 0.815; tdAUC_2yr_ avg = 0.826; C-index avg = 0.806.

**Table S3:** Bootstrap 95% confidence intervals of the held-out validation C-index at the primary landmarks (1000 resamples).

| **Landmark** | **n** | **Events** | **C-index** | **95% CI** |
| --- | --- | --- | --- | --- |
| t = 6 months | 111 | 17 | 0.793 | 0.713–0.882 |
| t = 12 months | 99 | 10 | 0.819 | 0.709–0.913 |

**Table S4:** Benchmark comparison on the common held-out validation set (n = 128). For each method, the fold-specific model with the highest C-index on the held-out split is shown. Best value in each column is shown in bold.

| **Model** | **Features** | **C-index** | **tdAUC_1yr_** | **tdAUC_2yr_** |
| --- | --- | --- | --- | --- |
| **Proposed** | **Full** | **0.806** | **0.815** | **0.827** |
| DeepSurv | Full | 0.733 | 0.745 | 0.737 |
| Cox-MLP | Full | 0.699 | 0.721 | 0.757 |
| ElasticNet | Full | 0.658 | 0.753 | 0.711 |
| Cox-MLP | Clinical | 0.707 | 0.760 | 0.711 |
| RSF | Clinical | 0.707 | 0.727 | 0.670 |
| GBSA | Clinical | 0.684 | 0.676 | 0.690 |
| ElasticNet | Clinical | 0.670 | 0.711 | 0.670 |
| GBSA | Omics | 0.672 | 0.647 | 0.688 |
| ElasticNet | Omics | 0.665 | 0.627 | 0.690 |
| Cox-MLP | Omics | 0.646 | 0.584 | 0.654 |

**Table S5:** External reduced-input benchmark on the GSE24080 cohort at the baseline landmark (t=1 month, n=507, 158 events).

| **Model** | **Features** | **C-index** | **tdAUC_1yr_** | **tdAUC_2yr_** |
| --- | --- | --- | --- | --- |
| **Proposed (Student)** | **Image + Clinical** | **0.672** | **0.740** | **0.721** |
| DeepSurv | All | 0.645 | 0.680 | 0.671 |
| Cox-MLP | Clinical | 0.628 | 0.666 | 0.649 |
| RSF | Clinical | 0.610 | 0.665 | 0.633 |
| GBSA | Clinical | 0.592 | 0.620 | 0.619 |
| Cox-MLP | All | 0.559 | 0.621 | 0.550 |
| Cox-MLP | Omics | 0.497 | 0.500 | 0.483 |
| GBSA | Omics | 0.481 | 0.519 | 0.476 |

**Note.** For external transfer, each benchmarked model used the fold-specific checkpoint selected on the common held-out CoMMpass validation split. The evaluation was performed without any use of GSE24080 for model or threshold selection.

**Table S6:** Multi-landmark external reduced-input evaluation on GSE24080 using the distilled student model. Performance at each landmark was evaluated independently; n decreases at later landmarks as patients with shorter follow-up are excluded from the at-risk set.

| $\boldsymbol{t}$ **(months)** | **n** | **C-index** | **tdAUC_1yr_** | **tdAUC_2yr_** |
| --- | --- | --- | --- | --- |
| 1 | 507 | 0.672 | 0.740 | 0.721 |
| 3 | 501 | 0.667 | 0.755 | 0.695 |
| 6 | 490 | 0.663 | 0.716 | 0.705 |
| 9 | 479 | 0.670 | 0.755 | 0.712 |
| 12 | 468 | 0.649 | 0.710 | 0.669 |

**Table S7:** Available-component ISS + baseline LDH comparison (not standard R-ISS). The ISS + baseline LDH score was formed as a standardized additive combination of the ISS stage and the standardized baseline LDH value, because assay-specific LDH upper-limit-of-normal reference ranges were not available; it did not improve over ISS alone (cf. main-text Table 2) and is reported here for completeness.

| **Landmark** | **n** | **C (proposed)** | **C (ISS+LDH)** | **ΔC** | **95% CI** | **p** |
| --- | --- | --- | --- | --- | --- | --- |
| t = 6 months | 100 | 0.800 | 0.589 | +0.211 | 0.081 to 0.362 | 0.002 |
| t = 12 months | 88 | 0.806 | 0.660 | +0.146 | 0.004 to 0.286 | 0.044 |

**Table S8:** Mean C-index improvement of the proposed model over each baseline averaged across the two primary landmarks (paired bootstrap, 1000 resamples).

| **Comparator** | **C proposed**  **(mean)** | **C comparator (mean)** | **ΔC**  **(mean)** | **95% CI** | **p** |
| --- | --- | --- | --- | --- | --- |
| ISS | 0.811 | 0.688 | +0.123 | 0.048 to 0.203 | < 0.001 |
| ISS + baseline LDH | 0.803 | 0.624 | +0.178 | 0.076 to 0.272 | 0.002 |
| Landmark Cox | 0.806 | 0.593 | +0.213 | 0.104 to 0.343 | < 0.001 |
| Landmark RSF | 0.806 | 0.657 | +0.149 | 0.033 to 0.264 | 0.010 |

**Note:** this pools the t = 6 and t = 12 landmark-specific risk sets, which share patients and are not independent; the p-values are therefore optimistic, and this table is a descriptive summary alongside, not in place of, main-text Table 2.

**Table S9:** Repeated five-fold cross-validation under three independent random seeds, summarizing run-to-run stability of the held-out C-index.

| **Seed** | **C-index** | **SD across folds** | **tdAUC_1yr_** | **tdAUC_2yr_** |
| --- | --- | --- | --- | --- |
| 42 | 0.773 | 0.024 | 0.789 | 0.798 |
| 1 | 0.749 | 0.032 | 0.758 | 0.788 |
| 2 | 0.770 | 0.024 | 0.787 | 0.787 |
| Aggregate | 0.764 | 0.013 | 0.778 | 0.791 |

**Table S10:** Parameter sensitivity of the proposed model on the held-out validation set. C-index and 1-year tdAUC are shown as each hyperparameter is varied around its default; no training instability or NaN losses were observed.

| **Parameter** | **Value** | **C-index** | **tdAUC1yr** |
| --- | --- | --- | --- |
| Image embedding dimension | 48 | 0.766 | 0.806 |
| Image embedding dimension | 64 | 0.773 | 0.800 |
| Fusion dropout | 0.25 | 0.795 | 0.832 |
| Fusion dropout | 0.35 | 0.777 | 0.814 |
| Fusion dropout | 0.45 | 0.807 | 0.824 |
| Auxiliary image-loss weight | 0.10 | 0.809 | 0.833 |
| Auxiliary image-loss weight | 0.15 | 0.781 | 0.810 |
| Auxiliary image-loss weight | 0.20 | 0.806 | 0.833 |
| Auxiliary tabular-loss weight | 0.05 | 0.794 | 0.821 |
| Auxiliary tabular-loss weight | 0.10 | 0.793 | 0.801 |

**Table S11:** Calibration stability across the five cross-validation folds (mean absolute calibration error of predicted vs Kaplan-Meier observed survival). Aggregation differs from Figure S3 (a single-fold quantile-bin calibration at t = 12 months); values are therefore not directly comparable.

| **Landmark** | **Horizon** | **MAE (mean)** | **MAE (SD)** | **Folds** |
| --- | --- | --- | --- | --- |
| t = 6 months | 1-year | 0.056 | 0.010 | 5 |
| t = 6 months | 2-year | 0.075 | 0.024 | 5 |
| t = 12 months | 1-year | 0.067 | 0.010 | 5 |
| t = 12 months | 2-year | 0.066 | 0.015 | 5 |

**Table S12:** Schoenfeld residual test of the proportional-hazards assumption for the model risk score at the primary landmarks. No significant evidence of a PH violation was observed; given the small event counts this is a diagnostic rather than a definitive check.

| **Landmark** | **n** | **Events** | **Test statistic** | **p-value** |
| --- | --- | --- | --- | --- |
| t = 6 months | 111 | 17 | 0.414 | 0.520 |
| t = 12 months | 99 | 10 | 0.038 | 0.846 |

**Supplementary Figures**

**Figure S1:** Training convergence for the selected fold (fold 2). (A) Negative Cox partial log-likelihood loss over training epochs. (B) Training and validation C-index trajectories; the vertical dashed line marks the best epoch (epoch 62) as determined by the EMA-smoothed tdAUC1yr monitor. (C) Validation tdAUC1yr averaged across primary landmarks ($t=6,12$ months), used as the early stopping criterion. (D) Validation Brier score (1-year horizon); the red line shows the 5-epoch rolling mean. Validation metrics were computed using model-weight exponential moving average (Polyak averaging, decay 0.998).

*Alt-text: Four-panel training-convergence plot for the selected fold: declining Cox partial-likelihood loss over epochs, training and validation C-index curves with the best epoch (62) marked, validation 1-year AUC used as the early-stopping monitor, and validation Brier score, together indicating stable convergence without overfitting.*

**Figure S2:** Benchmark comparison across five-fold cross-validation (mean $\pm$ SD). C-index, tdAUC_1yr_, and tdAUC_2yr_ are shown for the proposed model and all baselines, grouped by input feature set. Error bars represent one standard deviation over five folds computed on matched stratified splits. This figure complements main-text Table 1 (fold-level means) and Figure 3 (best-fold point estimates).

*Alt-text: Grouped bar chart of five-fold cross-validation C-index and 1- and 2-year time-dependent AUC (mean ± SD) for the proposed model and all baselines by input feature set, with the proposed model leading on every metric.*

**Figure S3:** Calibration assessment at the $t=12$ month landmark on the held-out validation set ($n=99$). (A) Predicted versus Kaplan–Meier observed 1-year survival probability (mean absolute error, MAE $=0.118$). (B) Predicted versus Kaplan–Meier observed 2-year survival probability (MAE $=0.088$). Each point represents a patient-level predicted–observed pair obtained by stratifying predicted survival probabilities into quantile bins and computing the corresponding Kaplan–Meier estimate within each bin. The diagonal dashed line indicates perfect calibration; solid lines show locally weighted regression fits. The lower MAE at the 2-year horizon reflects the model’s tendency toward conservative (higher) survival probability estimates that align more closely with observed outcomes over longer follow-up.

*Alt-text: Two-panel calibration plot at the 12-month landmark: predicted versus Kaplan–Meier-observed 1-year and 2-year survival probabilities fall close to the diagonal (mean absolute error 0.118 and 0.088).*

**Figure S4:** Internal modality ablation on the CoMMpass development set (five-fold CV, mean $\pm$ SD). C-index, tdAUC_1yr_, and tdAUC_2yr_ are shown for each single-modality configuration and the full multimodal model. Error bars represent one standard deviation across five folds. Complements main-text Table 3.

*Alt-text: Grouped bar chart of five-fold cross-validation C-index and 1- and 2-year time-dependent AUC (mean ± SD) for each single-modality configuration and the full multimodal model, which performs best.*

**Figure S5:** Single-modality performance drop relative to the full multimodal model, expressed as $\Delta$C-index (five-fold CV mean). Each bar shows the reduction in C-index when the model is restricted to a single data source: laboratory trajectories only ($\Delta=-0.080$), drug history only ($-0.145$), DeepInsight image representation only ($-0.149$), and expression MLP baseline ($-0.177$). Longitudinal laboratory measurements exhibit the smallest performance drop, consistent with their central role in ISS/R-ISS staging.

*Alt-text: Bar chart of the C-index reduction when the model is restricted to one modality (labs only −0.080, drugs only −0.145, image only −0.149, expression MLP −0.177); the smallest drop for labs-only identifies longitudinal laboratory trajectories as the strongest single modality.*

**Figure S6:** Representative Integrated Gradients attribution maps on the DeepInsight gene expression images, grouped by model-predicted risk category. **(A)** Low-risk patients exhibit diffuse, low-magnitude attribution patterns. **(B)** High-risk patients show spatially concentrated, high-magnitude activations. **(C)** Difference map (high-risk minus low-risk) highlighting genomic regions with the largest risk-associated attribution shift; individual genes are annotated at peak locations by reverse-mapping pixel coordinates to gene identifiers via the pyDeepInsight coordinate matrix.

*Alt-text: Three-panel Integrated Gradients attribution maps on the DeepInsight gene-expression images: diffuse low-magnitude patterns for low-risk patients, spatially concentrated high-magnitude activations for high-risk patients, and a high-minus-low difference map with peak genes annotated.*

**Figure S7:** Over-representation analysis (ORA) of risk-increasing genes against the MSigDB Hallmark 2020 gene set collection. Risk-increasing genes were defined as those with positive mean Integrated Gradients attribution (i.e., higher expression associated with higher predicted log-hazard). Dot size is proportional to the gene ratio (number of overlapping genes / gene set size); colour indicates false discovery rate (FDR). Top enriched terms include Unfolded Protein Response, Epithelial–Mesenchymal Transition, mTORC1 Signaling, Glycolysis, and Angiogenesis.

*Alt-text: Dot plot of over-representation analysis for risk-increasing genes against the MSigDB Hallmark collection (dot size = gene ratio, colour = FDR); top enriched terms include Unfolded Protein Response, Epithelial–Mesenchymal Transition, mTORC1 Signaling, Glycolysis and Angiogenesis.*

**Figure S8:** Over-representation analysis (ORA) of protective genes against the MSigDB Hallmark 2020 gene set collection. Protective genes were defined as those with negative mean Integrated Gradients attribution (i.e., higher expression associated with lower predicted log-hazard). Top enriched terms include Estrogen Response Early, Unfolded Protein Response (with gene members distinct from those in Figure S7), Androgen Response, and Wnt/$\beta$-Catenin Signaling. The presence of UPR in both risk and protective sets reflects the dual role of endoplasmic reticulum stress in plasma cell biology.

*Alt-text: Dot plot of over-representation analysis for protective genes against the MSigDB Hallmark collection (dot size = gene ratio, colour = FDR); top enriched terms include Estrogen Response Early, Unfolded Protein Response, Androgen Response and Wnt/β-Catenin Signaling.*

**Figure S9:** Drug utilization patterns stratified by model-predicted risk group on the held-out CoMMpass validation set. Monthly administration rates are shown for bortezomib, carfilzomib, and immunomodulatory drugs (IMiDs). The high-risk group (death rate 34%) received higher carfilzomib and IMiD utilization across all time bins compared with the low-risk group (death rate 1%), consistent with intensified treatment for higher-risk disease. These patterns reflect indication bias inherent in observational treatment data rather than causal treatment effects.

*Alt-text: Line chart of monthly bortezomib, carfilzomib and IMiD administration rates by model-predicted risk group; the high-risk group received more carfilzomib and IMiDs across all time bins, reflecting indication bias in observational data rather than causal treatment effects.*

**Supplementary Methods**

**S1.1 Gene expression preprocessing**

This section expands on main-text §2.3.1 with full preprocessing parameters. From the bulk RNA-seq expression matrix of the CoMMpass development cohort, the 5000 genes with the highest cross-patient expression variance were retained, with variance computed on the training set only to prevent information leakage. The selected gene set was then embedded into a two-dimensional coordinate grid using $t$-SNE (perplexity 30, cosine distance) fitted exclusively on the training-set expression matrix; these coordinates were frozen and reused without refitting for all validation, held-out, and external cohort samples. Expression values were min–max normalized using training-set percentile statistics and converted to pixel intensities after clipping at the 1st and 99th percentiles to suppress outlier influence. When multiple genes mapped to the same pixel (an unavoidable consequence of projecting 5000 features into a $96\times96$ grid), the pixel intensity was set to the mean normalized expression of the mapped genes. For the external GSE24080 cohort, gene symbols were matched to the frozen training-set gene set (4834 of 5000 matched, 96.7%); the 166 unmatched genes were zero-filled at their frozen coordinates, representing an assumption of absent measurement rather than absent expression. Alternative imputation strategies (e.g., gene-neighbour mean imputation using $t$-SNE coordinate proximity) were not pursued and are left for future work.

**S1.2 Encoder and fusion architecture details**

This section expands on main-text §2.4.1 with full layer-level specifications. The complete teacher model contains $\sim1.6$M trainable parameters.

*Gene expression encoder*

The $96\times96\times1$ DeepInsight image is processed by a lightweight convolutional network comprising five convolutional blocks, each consisting of a $3\times3$ convolution, batch normalization, ReLU activation, and $2\times2$ max-pooling. The convolutional stack is followed by adaptive average pooling and a fully connected projection layer with dropout 0.3, producing a 64-dimensional image embedding ($IMAGE\_OUT\_DIM=64$). The encoder is fully trainable from the start, with its learning rate scaled by a factor of 0.1 relative to the base learning rate to preserve low-level filter stability during joint fine-tuning. Parameter count: $\sim1.0$M.

*Laboratory dual-stream Transformer encoder*

The laboratory encoder receives a sequence of length $t$ covering monthly time bins $1,\ldots,t$. At each time step $j\leq t$, the input feature vector concatenates the observed analyte values, the binary observation mask $m_{j}$, the per-analyte time since last observation (TSLO), and the any-observation flag. This vector is projected to dimension $d=128$, combined with learnable positional embeddings, and processed by a two-layer Transformer encoder (4 attention heads, feedforward dimension 256). A parallel missingness stream embeds only the observation-pattern features (masks, TSLO, any-observation flags) using an identical Transformer configuration but with an elevated dropout rate to discourage the model from memorizing idiosyncratic missingness patterns as a shortcut. The value stream and the missingness stream are combined through a gating layer that produces the final lab modality embedding. Causal attention masks restrict each token to attend only to bins within $[1,t]$, enforcing temporal integrity at the attention level.

*Drug encoder*

The drug encoder processes the three-class drug usage grid (bortezomib, carfilzomib, IMiDs) along with the analogous per-class TSLO features using a two-layer Transformer encoder (4 attention heads, embedding dimension $d=64$).

*Time embedding*

The prediction time $t$ is encoded as a learnable embedding of dimension 16, shared across all landmark times and broadcast to the fusion module.

*Gated fusion module*

Each modality embedding (image, lab, drug) is projected to a shared dimension $d=128$ and layer-normalized. A gate network—a two-layer MLP with hidden dimension 64 and dropout 0.30—takes as input the time embedding $e(t)$ concatenated with per-modality TSLO summaries and produces softmax-normalized modality weights. The fused embedding is the weighted sum of the three projected modality embeddings, followed by a fusion dropout of 0.35.

*Auxiliary supervision and modality dropout*

Two regularization mechanisms address modality collapse and deployment-time robustness. First, per-modality auxiliary Cox proportional hazards heads provide direct gradient supervision to the image encoder (loss weight $\lambda_{img}=0.15$) and to the tabular (lab + drug) stream (loss weight $\lambda_{tab}=0.10$), in addition to the fusion-level primary Cox head. Second, deploy-aware modality dropout (probability $p_{drop}=0.30$) jointly zeroes both clinical embeddings (laboratory and drug) during training, simulating the external deployment setting in which only the baseline gene-expression-derived image representation is available. The image stream is never dropped, reflecting that the gene expression image is the one modality guaranteed to be available at deployment.

**S1.3 Training strategy implementation details**

This section expands on main-text §2.5 with the implementation-level details of patient-level sampling, curriculum-weighted landmark sampling, and the optimization schedule.

*Patient-level sampling*

Training operates at the patient level rather than the (patient, $t$)-pair level. Each patient contributes exactly $K=3$ landmark samples per epoch, preventing over-representation of patients with longer follow-up (who would otherwise dominate the training signal under uniform pair-level sampling) and ensuring that every patient is seen a controlled number of times per epoch. This design stabilizes gradient estimates and reduces variance in the Cox partial likelihood, which is particularly sensitive to risk-set composition when the overall event rate is low (19.9% in the CoMMpass development set).

*Curriculum-weighted landmark sampling*

For each patient sample, the landmark month $t$ is drawn from a curriculum-weighted distribution that concentrates probability on early, event-rich landmarks during the first phase of training and progressively broadens toward uniform coverage of $t\in\{1,2,\ldots,18\}$ as training proceeds. This schedule ensures that the model first learns from the most informative risk-set compositions—where event density is highest and the gradient signal on the partial likelihood is strongest—before extending to sparser later landmarks. Together, patient-level sampling and curriculum-weighted landmark sampling define a sampling contract: every patient is seen equally often, and every landmark receives attention proportional to its current learning utility.

*Evaluation protocol*

The 624-patient CoMMpass development set was partitioned into five stratified folds for model training. Within each fold, checkpoint selection and early stopping were performed using fold-internal validation metrics only. After training, the resulting fold-specific models were each evaluated on a separate common held-out CoMMpass validation split ($n=128$) that was not used for parameter fitting in any fold. Cross-fold mean $\pm$ SD on this common held-out validation split are reported as the primary comparative performance summary (main-text Table 1; Supplementary Figure S2). For downstream visualization and representative held-out analyses, a selected fold (fold 2) is additionally reported, chosen by the highest mean tdAUC_1yr_ across the primary landmarks ($t=6,12$ months) on the common held-out validation split. This selected fold was used for the landmark-wise plots, Kaplan–Meier stratification figures, and teacher-to-student transfer analyses (main-text Figures 2–5; Supplementary Tables S2–S6). For benchmark transfer to the external GSE24080 cohort, each benchmarked method was represented by its own fold-specific model selected on the same common held-out CoMMpass validation split. The external cohort was used strictly for final external evaluation; no model selection, fold selection, checkpoint selection, threshold derivation, or hyperparameter tuning used any GSE24080 data.

*Optimization*

Training used the AdamW optimizer with a base learning rate of $1\times{10}^{-4}$, weight decay 0.01, and linear warmup over 30 epochs. A ReduceLROnPlateau scheduler (factor 0.5, patience 15 epochs) reduced the learning rate upon metric stagnation. Gradient norms were clipped at 1.0. Batch size was 48 with gradient accumulation over 4 steps, yielding an effective batch size of 192. This configuration was chosen to ensure that each Cox partial likelihood batch contains a sufficient number of events for stable gradient estimation despite the low overall event rate.

*Early stopping and checkpoint selection*

Early stopping monitored the EMA-smoothed ($\alpha=0.35$) validation tdAUC_1yr_ averaged across primary landmarks ($t=6,12$ months), with patience 50 epochs and a minimum epoch gate of 60 per fold (maximum 600 epochs per fold). The minimum epoch gate ensures that the curriculum-weighted landmark sampling has completed its transition from early-landmark concentration to balanced coverage before checkpoint selection begins, preventing premature convergence to an early-landmark-biased solution. Model weight EMA (Polyak averaging, decay 0.998) was used for all validation and final evaluation steps.

*Image augmentation*

Image augmentation consisted of additive Gaussian noise ($\sigma=0.02$) and random erasing ($p=0.1$). Geometric augmentations (flips, rotations) were disabled because the $t$-SNE-derived spatial layout of the DeepInsight image lacks natural rotational or reflective symmetry: flipping or rotating the image would destroy the local co-expression neighbourhood structure that the convolutional encoder is designed to exploit.

**S1.4 Risk stratification threshold derivation**

For Kaplan–Meier risk stratification, predicted risk scores at each landmark were dichotomized into high- and low-risk groups. The threshold at each landmark was determined by maximizing the log-rank test statistic over a grid of candidate cut-points applied to the training-set risk score distribution. These training-derived thresholds were then applied without modification to the held-out validation set and the external cohort (GSE24080), ensuring that no information from the evaluation data influenced the group assignment.

**S1.5 Knowledge distillation configuration**

Student input: DeepInsight gene-expression-derived image ($96\times96\times1$) + five baseline clinical features (HGB, CREAT, ALB, LDH, $\beta_{2}$M). Architecture: LightweightCNN (identical topology to the teacher image encoder) + a small clinical MLP; total $\sim1.0$M parameters. The student image encoder was initialized from the corresponding teacher weights to warm-start the transfer. Composite training loss: $L_{student}=\alpha_{surv}\cdot l_{Cox}+\alpha_{out}\cdot L_{KD}+\alpha_{feat}\cdot L_{align}$, with $\alpha_{surv}=0.3$ (Cox partial likelihood on student outputs), $\alpha_{out}=0.5$ (output-level distillation of teacher log-hazard predictions), and $\alpha_{feat}=0.2$ (feature-level cosine alignment between teacher and student fusion embeddings). Student learning rate: $3\times{10}^{-4}$. Distillation was performed at a single landmark $t=1$ month, matching the baseline availability constraint of the GSE24080 target cohort. Best checkpoint: epoch 86 of 146 (EMA-smoothed tdAUC_1yr_ $=0.831$ on the internal validation split).

**S1.6 Feasibility of standard prognostic baselines**

Formal R-ISS and R2-ISS staging require cytogenetic and translocation status, in particular del(17p), t(4;14) and t(14;16), together with an elevated-LDH criterion. In the public GDC CoMMpass data, the structural-variant and gene-fusion files needed to derive these translocations were controlled-access, with no open-access files available for direct parsing; in addition, the processed laboratory files do not contain assay-specific upper-limit-of-normal reference ranges, so a formal thresholded elevated-LDH criterion cannot be applied. We therefore report ISS and an available-component ISS + baseline LDH score (Supplementary Table S7) as conservative baselines representing the reconstructable components of R-ISS, and we explicitly label the latter as not standard R-ISS.

The GEP70 and EMC-92 signatures are full-transcriptome classifiers with platform-specific probe definitions. We checked the overlap between the published GEP70 and EMC-92 signature genes and the processed expression features available for this cohort and found the coverage insufficient for a faithful reconstruction; EMC-92 additionally relies on a proprietary coefficient set. Evaluation against complete cytogenetics-based staging and full-transcriptome signatures, in cohorts where these are available, is an important direction for future work.

**Supplementary Discussion**

**Gene-level biological interpretation**

This section expands on Section 3.7 of the main text, providing gene-by-gene functional elaboration for the top-ranked risk-associated and protective genes identified by Integrated Gradients attribution on the DeepInsight image representation branch. The discussion below is intended as biological context rather than mechanistic claim: the attribution analysis identifies correlative transcriptomic patterns learned by the model, not causal drivers of disease progression.

*Top risk-associated genes*

**UBE2Q1** had the highest mean Integrated Gradients attribution among risk-increasing genes. UBE2Q1 encodes a ubiquitin-conjugating (E2) enzyme within the ubiquitin–proteasome system (UPS), a pathway central to plasma cell biology and to the therapeutic activity of proteasome inhibitors including bortezomib and carfilzomib [1,2]. The prominence of UBE2Q1 is consistent with the dependence of myeloma plasma cells on proteasomal protein turnover to manage the high immunoglobulin secretory load characteristic of the disease.

**ADAR**, encoding an RNA adenosine deaminase responsible for A-to-I RNA editing, has been reported to be overexpressed in myeloma and to drive aberrant hyperediting of the transcriptome, conferring oncogenicity and marking poor prognosis [3].

**P4HB** encodes protein disulphide isomerase, a resident endoplasmic reticulum chaperone that catalyses disulphide bond formation during immunoglobulin folding. The prominence of P4HB aligns with the marked dependence of myeloma plasma cells on endoplasmic reticulum homeostasis and their vulnerability to unfolded protein response disruption induced by proteasome inhibitor therapy [4].

**PRPF8**, a core component of the U5 small nuclear ribonucleoprotein particle, anchors the catalytic core of the spliceosome. Its appearance among top risk genes is consistent with emerging evidence that RNA splicing is dysregulated in multiple myeloma and that the spliceosome itself represents a therapeutic vulnerability in the disease [5].

*Top protective genes*

**SPEN** encodes a transcriptional repressor that modulates the Notch signaling pathway and has broad roles in chromatin and lineage regulation. **RNF169** is a DNA damage response factor that restricts non-homologous end-joining in favour of homologous recombination-based repair. The negative attribution of these genes suggests that higher expression is associated with lower predicted log-hazard in the model; both roles are mechanistically plausible in the context of genome maintenance and lineage stability in myeloma plasma cells, but the association is correlative and should not be read as a direct protective mechanism.

*Pathway-level convergence*

At the pathway level, pre-ranked GSEA on MSigDB Hallmark gene sets (main-text Figure 6D) and ORA on the separated risk-increasing and protective gene sets (Supplementary Figures S7 and S8) converged on several recurring themes: the Unfolded Protein Response and mTORC1 Signaling among risk-enriched terms, and Interferon Alpha Response among risk-associated pathways. Interferon Alpha Response enrichment among risk genes is notable in light of evidence that type I interferon signaling can promote myeloma-associated immunosuppression and disease progression via regulatory T-cell expansion [6]. Collectively, the gene-level and pathway-level findings recover axes of myeloma biology that are independently established in the literature, providing a form of sanity check on what the model has learned from the gene expression image branch, without themselves constituting new biological discovery.

**References**

1. Lub S, Maes K, Menu E, et al. Novel strategies to target the ubiquitin proteasome system in multiple myeloma. Oncotarget 2016; 7:6521–6537

2. Cao B, Mao X. The ubiquitin-proteasomal system is critical for multiple myeloma: Implications in drug discovery. Am. J. Blood Res. 2011; 1:46–56

3. Teoh PJ, An O, Chung T-H, et al. Aberrant hyperediting of the myeloma transcriptome by ADAR1 confers oncogenicity and is a marker of poor prognosis. Blood 2018; 132:1304–1317

4. Obeng EA, Carlson LM, Gutman DM, et al. Proteasome inhibitors induce a terminal unfolded protein response in multiple myeloma cells. Blood 2006; 107:4907–4916

5. Huang H-H, Ferguson ID, Thornton AM, et al. Proteasome inhibitor-induced modulation reveals the spliceosome as a specific therapeutic vulnerability in multiple myeloma. Nat. Commun. 2020; 11:1931

6. Kawano Y, Zavidij O, Park J, et al. Blocking IFNAR1 inhibits multiple myeloma-driven Treg expansion and immunosuppression. J. Clin. Invest. 2018; 128:2487–2499
